# A model linking clinical workforce skill mix planning to health and health care dynamics

**DOI:** 10.1186/1478-4491-8-11

**Published:** 2010-04-30

**Authors:** Keith Masnick, Geoff McDonnell

**Affiliations:** 1 School of Public Health and Community Medicine, University of New South Wales, Kensington, Australia; 2 Centre for Health Informatics, University of New South Wales, Kensington, Australia

## Abstract

**Background:**

In an attempt to devise a simpler computable tool to assist workforce planners in determining what might be an appropriate mix of health service skills, our discussion led us to consider the implications of skill mixing and workforce composition beyond the 'stock and flow' approach of much workforce planning activity.

**Methods:**

Taking a dynamic systems approach, we were able to address the interactions, delays and feedbacks that influence the balance between the major components of health and health care.

**Results:**

We linked clinical workforce requirements to clinical workforce workload, taking into account the requisite facilities, technologies, other material resources and their funding to support clinical care microsystems; gave recognition to productivity and quality issues; took cognisance of policies, governance and power concerns in the establishment and operation of the health care system; and, going back to the individual, gave due attention to personal behaviour and biology within the socio-political family environment.

**Conclusion:**

We have produced the broad endogenous systems model of health and health care which will enable human resource planners to operate within real world variables. We are now considering the development of simple, computable national versions of this model.

## Background

The current health workforce planning literature is very much concerned with discussions and suggestions as to the optimal composition of a health workforce, but there has been remarkably little by way of tools to assist the planner in actually determining what might be an appropriate mix of skills and personnel [1]. WHO has recently provided a wide ranging discussion on the concept of 'Ten Steps To System Thinking' to look at strengthening health systems through the use of systems thinking [2].

However we have been attempting to devise a simple computable do-it-yourself tool which would help in drawing up and examining the staffing, service and costing implications of alternative skill mix scenarios. The scenarios would reflect different mixes of personnel categories, the shifting of tasks from one category of personnel to another, the substitution of one type of worker with another and the possible creation of new categories of health worker where this appeared to be desirable [3-6]. The planner would then have a repertory of alternative scenarios from which to select the most appropriate choice.

Our quest for such a tool has taken us well beyond the simple 'stock and flow' type planning approach which has been widely used in health workforce planning, which generally focussed on one or other particular category of health service personnel, most commonly doctors or nurses, and less frequently on dentists, pharmacists, optometrists, laboratory and medical imaging personnel, physiotherapists, speech therapists, community health workers, other clinical personnel groups. Focus on workers in managerial, administrative, engineering, housekeeping and other support personnel categories has only been occasional. We realized that we were not just thinking about a 'workforce problem'; rather we were confronting a 'dynamic system' problem [7,8] in which there are feedback and delays between decision-making and implementation.

Previously, Birch et al.'s needs-based analytic framework [9] had taken a step towards addressing more complexity by including services, epidemiology and demography in human resource planning. On the supply side, WHO [10] had proposed a six block model which incorporates technology, information and governance.

Our discussions led to the possibility of drawing a structural map of a health system, based on a synthesis of these two approaches, showing the interactive connections between its major components, which could be expanded at a later date to show the linkages between the tasks performed by a health workforce and the cadres of personnel that could supply those tasks. This paper presents an outcome of attempting to present such a map.

## Methods

We started modelling a system with three basic components - the population to be served, the clinical workforce to serve it, and the workload generated by both the population and the clinical workforce.

The population, ever changing in numbers and composition through the interplay of births, deaths and migration, is the source of people with 'health conditions', or more precisely 'ill-health conditions': some with disabilities, and some with disease, thus generating the need for clinical health care. For a range of reasons including personal choice, fears and prejudice, geographic and financial accessibility, perceived quality and acceptability of available services, some of that need will be manifest in demand for care and so constitutes part of the 'clinical workload' confronting the clinical workforce.

The clinical workload we are concerned with is the workload made up of the four essential functions of personal health service delivery: detection, identification, diagnosis, and management of health conditions and disability. These are functions involving person-to-person interaction between the affected person and one or more persons trained in at least one, but usually more of these four essential functions. The trained personnel in this interaction make up what we have called, and are generally recognised as, the 'clinical workforce'. 'Clinical workforce' should be understood here in its widest sense, to cover the range from the minimally-trained to the highest-skilled practitioners. Some readers may be uneasy with the absence of the word 'prevention' from our listing of clinical workload activities. The reason for this omission is our view that the preventive activities of clinicians in their face-to-face interaction with their patients, covering as they do all three levels of prevention--primary, secondary and tertiary--can well be subsumed under the broad heading 'management of health conditions', as part of a clinician's 'time doing work'.

Although health system policy makers, planners and managers are faced with issues extending well beyond concerns relating to the clinical workforce, we have chosen to concentrate our attention on this composite group because clinical personnel constitute the most numerous personnel group and the most financially costly element in virtually every national health care delivery system across the world [11].

### The population and people with health conditions

#### 1. Determinants of population numbers and composition

Population numbers and composition--commonly described in terms of age, sex and ethnicity--are, at the first level of analysis, the outcome of three processes: birth, death and migration [12]. These processes reflect the dynamic interaction of many factors, some relating to human genetics and human behaviour, others being responses to environmental influences beyond human control. Our interest is focussed on a particular group within a population at large, the group of people with what we have referred to as 'health conditions'.

#### 2. People with health conditions

Listings of classifiable and classified diseases and disabilities which may affect human beings--for example the entities listed in the items listed in the International Statistical Classification of Diseases and Related Health Problems,10^th ^Revision, Version for 2007--run into thousands of items [13]. For workforce planning purposes very basic groupings such as 'acute', 'chronic', 'life threatening' and 'requiring short-term or long-term institutional or ambulatory care' are generally sufficient. Many of the people with health conditions need clinical care, but, as we noted earlier, not all of them seek such care--we are concerned in our modelling here with those who do, since this expressed demand for care significantly determines the size and nature of the 'clinical workload'. We are well aware that this stock of people in need is not static but is affected by a wide range of elements, such as availability, changes in technology and government policies, and personal and cultural perceptions of services.

#### 3. Impact of 'non-clinical' preventive activity and 'alternative medicine'

Of course, 'non-clinical' preventive activity in its many and varied forms will have an important role in determining the number of people with health conditions, and the nature of those conditions. Substantial numbers of people in the 'with health conditions' group may seek or receive 'clinical care' from practitioners of 'alternative medicine' or other forms of clinical intervention. Our model does not take these factors into account, since we are concerned with planning relating to the size and composition of what we have chosen to regard as the 'professional' clinical health workforce.

### The clinical workload

We have discussed the determinants of the size and composition of the expressed demand for clinical care and we now need to express that demand in workload terms: what are the nature and volume of the inputs and processes required to meet the demand?

Previously we identified the essential processes involved in clinical care as detection, identification, diagnosis and treatment of disease and disability. The essential inputs to these processes can be identified as people expressing their demand for clinical care, the clinical health workforce and medical technology. Together these three elements determine the output and subsequent outcome of the service provided by the clinical sector of the health system.

### The clinical workforce

The clinical workforce of our concern is comprised principally of:

▪ 'Doctors' - medical practitioners who have graduated from a medical school on the completion of generally four to six years' training followed by one or more years of internship, and in the case of specialists several more years of advanced specialist training.

▪ Other professional health workers (e.g. dentists and pharmacists) who have usually completed four to six of university training.

▪ The range of trained nursing personnel.

▪ Second tier medical practitioners referred to variously by such titles as assistant medical officers, auxiliary medical officers, clinical officers, health officers, health extension officers, non-physician clinicians--generally having completed at least three years' training.

▪ Personnel with minimal training such as community health workers and medical aids.

### Current mental and spreadsheet stock-flow models

**Figure 1:**
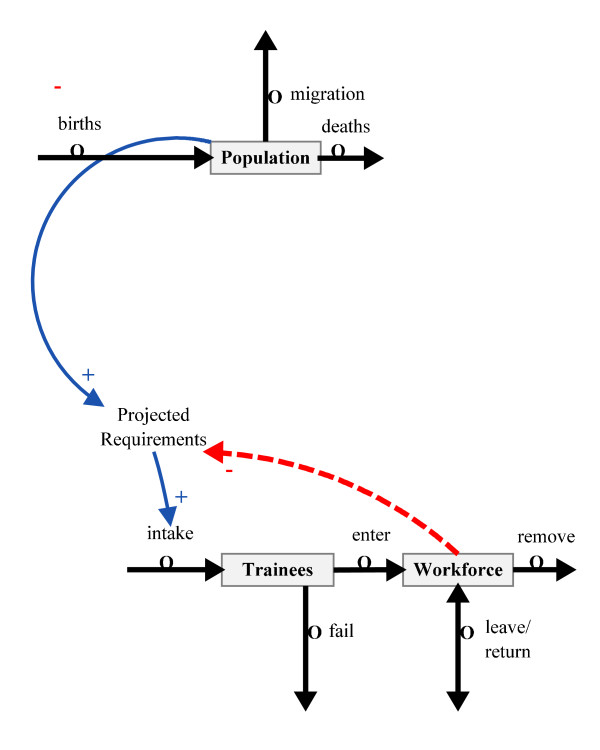
**Simple stock flow and population ratio model closing the gap between workforce and projected population requirements**. Note: The conventions of systems dynamics mapping are followed in this and subsequent figures. Interactions notated as  indicate that as the source increases the destination also increases. The dotted lines notated as  indicate that an increase at the source decreases the destination. For example, an increase in the population increases the projected requirements which increase the student intake. However, increased graduates will decrease the projected requirements for more personnel. Regulators to flows in and out of the system are indicated by .

A stock-and-flow approach to clinical workforce planning within a health system is essentially a numbers game. On the input side, contributing to the workforce stock, we have the number of students taken into training and the graduates subsequently employed in the health system. This training flow is supplemented by the number of clinicians previously trained but currently outside the health system who are recalled into employment, as well as the number of personnel trained elsewhere but imported to fill posts within the health system. On the exit side we have personnel who resign, retire, are re-assigned to non-clinical work, those who leave due to health reasons or are dismissed from the system, and those who die while in employment. This basic model of the main elements of the human resources subsystem is shown as Figure 1. The basic model could be opened up at a later date to include finer points such as recruitment and retention.

In Figure 1, 'projected requirements', in terms of personnel numbers, is based on an agreed number of doctors, dentists, nurses and midwives, etc., per population. Any difference between the current workforce and requirements results in an adjustment to intakes of trainees if the funds and support are available, or there are changes in overall workforce due to redundancy, retirement or migration.

### Clinical service workforce

**Figure 2:**
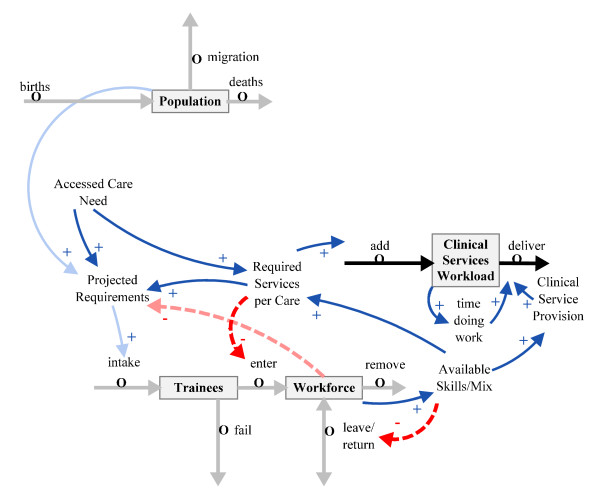
**Linking workforce to skill mix and clinical work**.

Regarding the volume and nature of the clinical services workload, this is determined by the accessed need generated by people in the population with health conditions. Planning for an effective and efficient clinical workforce calls for attention not simply to numbers, but to skill mix within the workforce, retention of trained personnel, worker time pressures, skills gaps effects on productivity, and appropriate deployment of clinical services enabling staff to do their work effectively. These aspects are added to Figure 1 as *Available skills mix *to create Figure 2.

**Figure 3:**
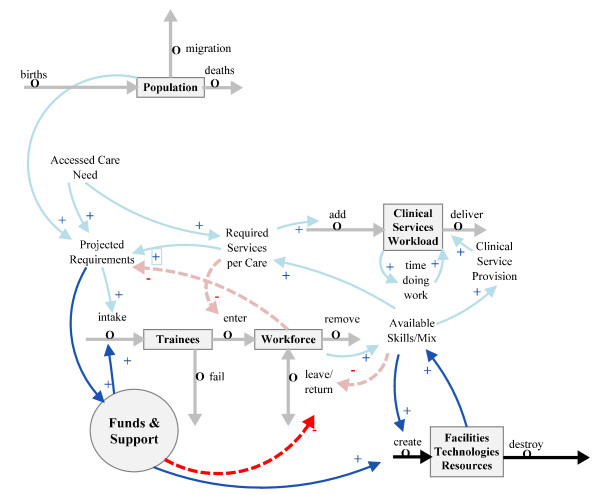
**Addition of resources and funds and support subsystems**.

A more complete supply side picture (Figure 3) includes a resource subsystem 'Facilities, Technologies and Resources', which encompasses all facilities and technologies, and also a financial subsystem 'Funds and Support', containing funding and the political support relevant to the clinical professionals' ability to provide clinical care.

It should be noted that in the diagram 'Funds & Support' is represented as a circle rather than a stock. The circle is used to depict the fact that this is a collection of individual payers and links the model to the vast literature that depicts the health system as a contested arena of conflicting interests [14,15]. The agents involved in this contest are usually referred to as payers, providers and patients. In addition, regulators or 'governors', people who use policies, power or other governance mechanisms (including managers and administrators), can be considered as other individual 'players' in the contest.

### Clinical care microsystems

**Figure 4:**
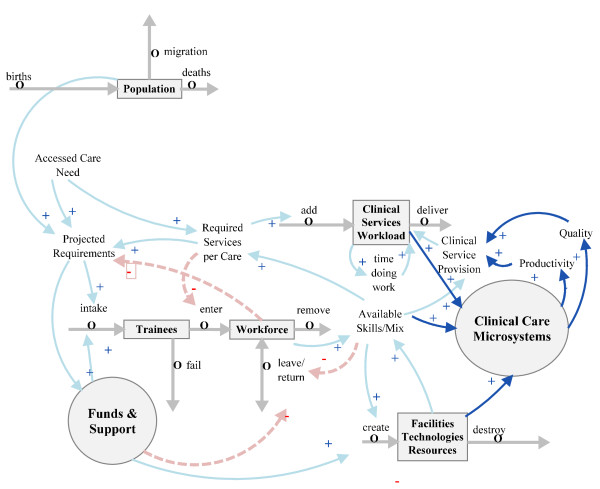
**Addition of clinical care microsystem agency**.

The basis of clinical care is the interactions among patients, healers and carers. This is often described in a more technical sense as the clinical microsystem, the way the care team, including the patient, work together to perform clinical work [16]. This is now seen as a complex socio-technical system with great potential for both superior results and catastrophic errors [17]. 'Clinical Care Microsystems' is added to the model in Figure 4, again with a circle, to represent the clinicians as individual agents. The clinical tasks of informing, deciding, acting and communicating are performed by interactions among the agents, who are constrained or enabled by the structural environment [18]. The effectiveness and efficiency of 'Clinical Service Provision' reflect the productivity and quality of work within 'Clinical Care Microsystems'.

### Linking clinical work to the rest of healthcare

Clinical work is performed by health professionals in institutional patient care settings. These include hospitals, health professionals' offices, primary health and community care clinics, outreach clinics, residential care institutions, and patients' homes. Patients in care enter and exit the various institutions that are provided in the facilities, service configurations and 'models of care' provided at the macro-level through the sectoral structure of health care.

**Figure 5:**
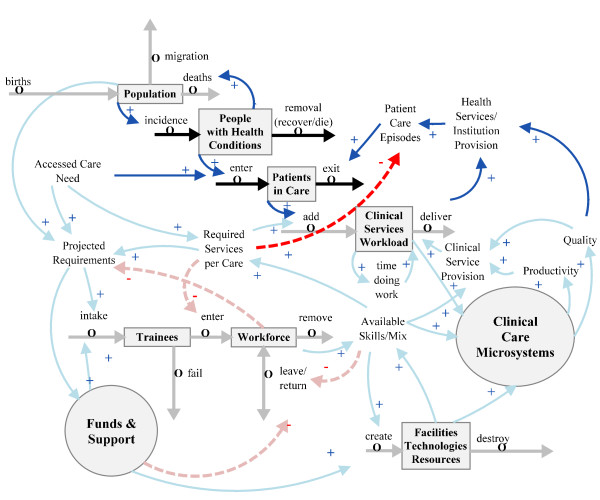
**Linking clinical work to population via patient flows and people with health conditions**.

Reflecting in greater detail the linkage between population and clinical workload [19,20], we interposed the two additional stocks of 'People with Health Conditions' and 'Patients in Care' between population and clinical workload (Figure 5).

'People with Health Conditions' includes people with acute infectious diseases [21], those with chronic disease conditions [22-24] and victims of trauma.

The 'Patients in Care' stock depicts those people contracted to a care institution to receive clinical services. In our model they are considered as patient care episodes, since this is the way health outputs or service activity is measured [25]. In a continuum of care, these episodes can last from brief care institution interactions between patient and healer/carer to a lifetime of chronic care, depending on the purpose of the model.

### Impacts of healthcare outputs on the population

**Figure 6:**
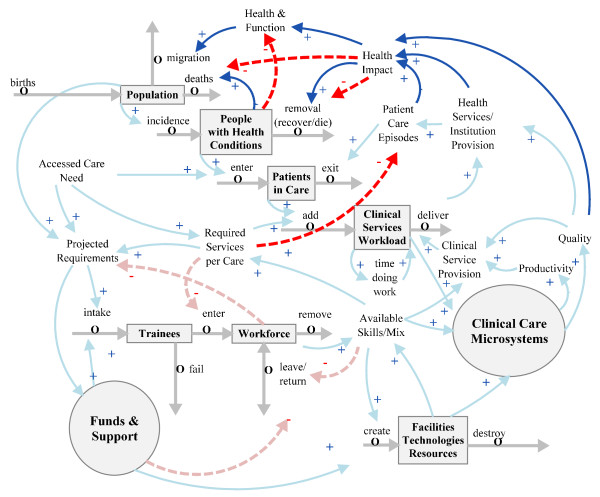
**Linking health impacts to population and people with health conditions**.

Patient care episodes should have measurable impacts on the health and function (that is, disease and disability prevalence) of the population [26]. Closing the loop to show how 'Health Impact' and 'Health and Function' affect the inflows and outflows of 'Population' stock is illustrated in Figure 6. These effects can be mediated by recovery, change in mortality and morbidity or in functional status, or any other factor which can affect health-related quality of life.

### Healthcare within the overall social structure

**Figure 7:**
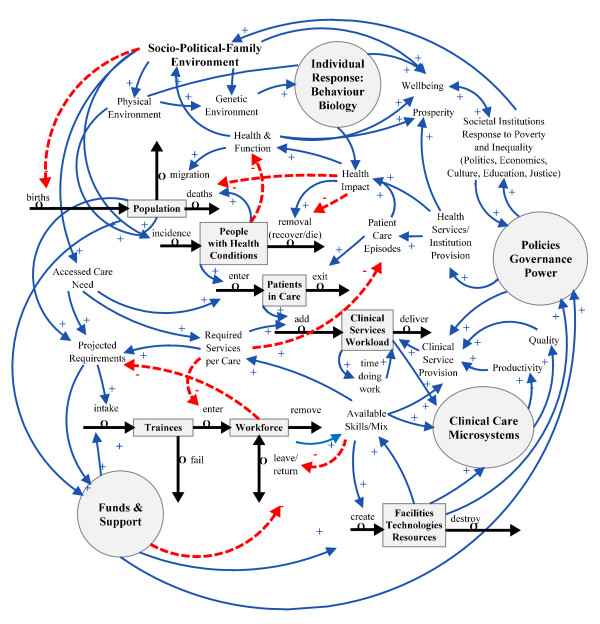
**Full scope depiction of health and healthcare dynamics**.

The many other factors that affect health and function include the whole of our human and physical environment, including the social determinants of health. A popular framework for representing the dynamics of health and wellbeing is the Evans, Barer, Marmor Field Theory of Health [26,27]. To complete the link between healthcare and the rest of the social system, we also need to add the broader governance structures that link health care values to the rest of the social institutional structures that affect citizens. The representation used here in Figure 7, 'Individual Response: Behaviour and Biology' centres on the individual as a social being and borrows heavily from the Structure-Agency Sociology theory popularised in the United Kingdom by Giddens [28] and identified as the philosophical foundation of the system dynamics method by Lane and Huseman [18,29]. Again the individual person is represented here as a circle, an individual agent. Much of the new work in systems biology and systems medicine occurs within the body of this agent [30]. In this area of research, the person is represented as a dynamic network of genetic information interacting with the environment through multiple scales, from the protein molecule to the cell to the organ to the body to the external world. Future management of health may involve preventing and managing the perturbation of these networks by disease [31,32]. The addition of the concept of 'Policies, Governance and Power' explicitly links the scope of the dynamics of health and health care to the political process within and outside healthcare, and also influences the individual's socio-political family environment.

Figure 7 represents our current depiction of the major components of the dynamic complexity of health and healthcare.

## Results

John Muir remarked, "When we try to pick anything by itself, we find it hitched to everything else in the universe" [33].

From the discussion on how best to manage the appropriate skills mix of personnel in the clinical workforce, we have developed a broad endogenous systems model of the dynamic complexity of health and healthcare.

This may provide the basis for exploring how the interactions among health policy, clinical practice, workforce and technology deliver efficient, equitable and effective healthcare services that produce healthier populations.

## Conclusion

We have set out to widen the breadth of human resource planning in order to capture the dynamic and complex nature of planning and the notion that human resources are but one dependant aspect of health services which itself is only one part of the whole interactions of people and their organisations. We have produced the broad endogenous systems model of health and health care described as a basis for a newer approach to human resource planning. We are now considering the development of simple, computable national versions of this model. However, this broader model raises many questions that our later research hopes to answer. One vital question for instance is whether our modelling is applicable to developing countries, where data may be sparse and the resulting quantitative structure may not lend itself to sufficient accuracy in order that sensitivity analysis can be performed.

The advantages of dynamic modelling are that it can provide leadership, co-ordination and inform planning in a real world context.

Our discussion and modelling have taken us a long way from designing the simple computable do-it-yourself workforce planning tool that we originally had in mind. We are now considering the development of a quantitative simplified national model of 5-8 stocks at the national level with technology and supply/demand interactions, focussing on training new clinical professionals.

## Competing interests

The authors declare that they have no competing interests.

## Authors' contributions

KM and GM both participated in the creation and preparation of the manuscript and have seen and approved the final version.
